# 48-year-old with Altered Mental Status and Respiratory Failure: A Case Report

**DOI:** 10.5811/cpcem.2021.3.51331

**Published:** 2021-04-23

**Authors:** Justin E. Pile, Justina Truong

**Affiliations:** * Swedish Hospital, Northshore University HealthSystem, Department of Emergency Medicine, Chicago, Illinois; † Kingman Regional Medical Center, Department of Emergency Medicine, Kingman, Arizona

**Keywords:** Cardiorenal Syndrome, Respiratory Failure, Altered Mental Status, Sepsis, Case Report

## Abstract

**Introduction:**

The differential diagnosis for altered mental status and respiratory failure is broad. Careful physical examination, appropriate use of diagnostic tools, and accurate interpretation and correlation of test results are important for piecing together the puzzle of a patient with altered mental status that emergency physicians commonly face. In certain cases, such as this one, rapid diagnosis and management is crucial for improving patient morbidity and mortality.

**Case Presentation:**

A 48-year-old male with altered mental status and respiratory failure presented to the emergency department after being found unconscious on his porch. Vital signs were notable for temperature 105.5 °F, blood pressure 202/102 millimeters of mercury, pulse 126 beats per minute, respiratory rate 30 breaths per minute, and oxygen saturation 91% on room air. Physical examination revealed an obese male lying in bed awake in severe distress with labored breathing and unable to converse. His physical examination was significant for dry mucous membranes, tachycardia, and bilateral lower extremity 1+ pitting edema. He also appeared to have Kussmaul respirations with severe tachypnea, but his breath sounds were clear to auscultation bilaterally. On further examination, the patient appeared to have intravenous (IV) injection markings along his arms suggesting the possibility of IV drug use.

**Discussion:**

With limited history, the only context clues initially available to assist in the diagnosis were abnormal vital signs and physical examination. The patient was tachycardic, hyperthermic, hypertensive, hypoxic, and tachypneic with altered mental status; he eventually required endotracheal intubation for hypoxic respiratory failure. The complexity of his condition prompted a large list for the differential diagnoses. Toxidromes, endocrine abnormalities, infectious process, cardiac and/or renal etiologies, and neurological pathology such as a cerebrovascular accident were considered. In the case of this patient, urgent diagnosis and management was crucial to prevent further decompensation and improve his outcome.

## CASE PRESENTATION (Resident Presentation)

A 48-year-old male presented to the emergency department (ED) via ambulance after being found unconscious on his porch. The paramedics stated the patient had labored breathing and altered mental status but did answer a few questions appropriately. The patient denied any drug use, but emergency medical services had administered eight milligrams of naloxone to him prior to arrival to the ED. In the ED, additional history including a review of systems was limited secondary to the patient’s clinical condition.

Vital signs were as follows: temperature 105.5 degrees Fahrenheit (°F), blood pressure 202/102 millimeters of mercury, pulse 126 beats per minute, respiratory rate 30 breaths per minute, and oxygen saturation 91% on room air. Physical examination revealed an obese male lying in bed awake in severe distress with labored breathing and unable to converse. Due to patient distress, orientation could not be assessed. He was able to move all extremities and had no facial asymmetry, but a full neurological exam was limited secondary to clinical condition. His physical examination was significant for dry mucous membranes, tachycardia, and bilateral lower extremity 1+ pitting edema. He also appeared to have Kussmaul respirations with severe tachypnea but had breath sounds clear to auscultation bilaterally. On further examination, the patient appeared to have intravenous (IV) injection markings along his arms suggesting the possibility of IV drug use.

Laboratory and imaging evaluation in the ED included numerous diagnostic tests. Abnormal laboratory results were found within the complete blood count, comprehensive metabolic panel, lactic acid, lactate dehydrogenase, creatine kinase, C-reactive protein, procalcitonin, high-sensitivity troponin, B-type natriuretic peptide, D-dimer, international normalized ratio, fibrinogen, urinalysis, urine culture, blood cultures, arterial blood gas, and urine drug screen. An electrocardiogram (ECG), chest radiograph (CXR), computed tomography (CT) of the head without contrast, and CT of the chest, abdomen, and pelvis with contrast were ordered.

Unfortunately, the patient’s mental and respiratory status did not improve while in the ED and he was intubated and mechanically ventilated due to hypoxic respiratory failure. He was started on a propofol infusion for post-intubation sedation, which assisted in blood pressure reduction. Given his hyperthermia at 105.5°F, passive cooling was initiated, and judicious fluid administration was considered in light of possible congestive heart failure. Thus, a one-liter normal saline bolus was administered. Broad spectrum antibiotics including vancomycin and piperacillin/tazobactam were initiated as well. The patient was admitted to the intensive care unit (ICU).

## CASE DISCUSSION (Attending Discussion)

**Image 1:**
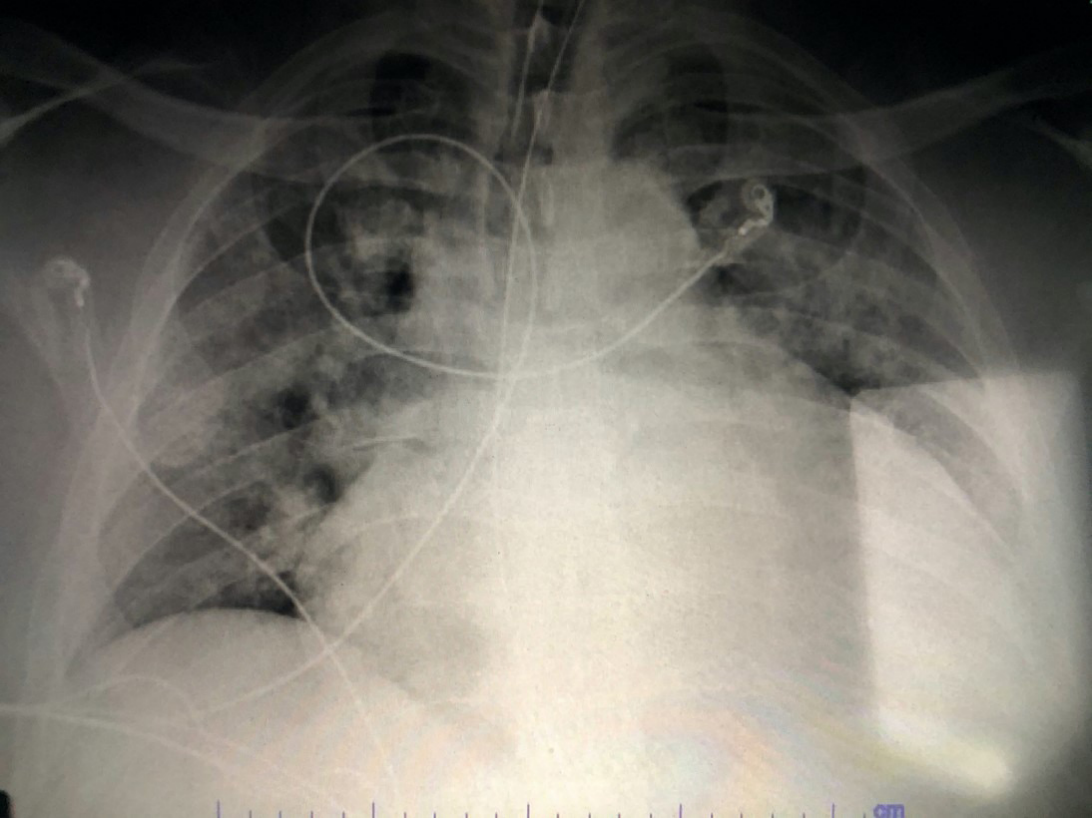
Anterior-posterior chest radiograph of a 48-year-old male with respiratory distress and altered mental status.

**Image 2:**
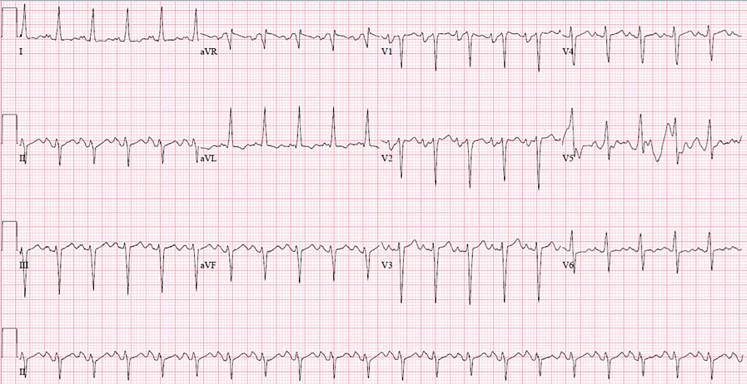
Electrocardiogram of a 48-year-old male with respiratory distress and altered mental status.

Based on the limited information, the differential diagnosis inferred from the patient’s presentation remained broad. The top considerations were infection, toxidrome, endocrine abnormality, or metabolic emergency. Based on the laboratory and imaging results presented below in Table 1 and Images 1 and 2, certain diagnoses were considered more or less likely. For example, due to the patient’s tachycardia, hyperthermia, and altered mental status, thyroid storm was considered in the differential diagnosis. No thyroid studies were ordered as there were other more likely causes of the patient’s presentation. Another endocrine abnormality considered was diabetic ketoacidosis, but this was less likely considering a blood glucose level in the high 100s, negative ketones in the urine, as well as a pH that was slightly alkaline. Examination showed no signs of major trauma or focal neurological deficits. Imaging of the head, chest, abdomen, and pelvis showed no acute fractures or hemorrhage, decreasing the chance of the patient suffering from pathology such as pneumothorax, internal bleeding, or an acute cerebral vascular accident.

The patient presented in obvious respiratory distress and had oxygen saturations of only 91% on arrival, making cardiopulmonary diagnoses high on our list of differential diagnoses. Given the pitting edema, respiratory failure, and the CXR (Image 1) showing cardiomegaly, new-onset heart failure was suspected. The patient also had an elevated troponin level, but no signs of ST-segment elevation on ECG as seen in Image 2. The troponin was likely elevated due to the patient’s significantly elevated blood pressure suggesting hypertensive emergency. His elevated white blood cell count, slightly elevated lactic acid level, and abnormal vital signs indicated severe sepsis likely from an infectious source with the most likely etiology an acute urinary tract infection, based on urinalysis results. Renal insufficiency was noted on laboratory evaluation as well, likely resulting from cardiac dysfunction or severe sepsis with multiorgan dysfunction.

Toxidromes or overdose were also considered. The patient had no reported or known medical history, so prescribed psychiatric medications were unlikely. Salicylate toxicity was considered given the patient’s respiratory alkalosis, pulmonary edema, hyperthermia, and altered mental status. However, initial salicylate levels were negative as were acetaminophen and ethanol levels. Alcohol withdrawal was considered as well due to the patient’s altered mental status and autonomic instability, but no other exam or history findings supported this. His urine drug screen was positive for methamphetamine, amphetamines, and cannabinoids. The physical exam findings were concerning for signs of IV drug use suggesting that this patient’s acute illness was possibly related to polysubstance abuse. Infective endocarditis was a consideration as well based on his fever, IV drug use, and likely new congestive heart failure. However, further diagnostic studies with echocardiogram and blood culture results would be needed to include or exclude this diagnosis.

Central nervous system infection was considered as well and further testing with lumbar puncture could be performed to assess for this. Given the patient’s presentation, vital signs, labs, and imaging findings, the conclusion could be made that he suffered from cardiogenic and septic shock. Sources of acute illness include urinary infection, possible endocarditis or bacteremia, and polysubstance abuse.

## CLINICAL DIAGNOSIS

Cardiorenal syndrome (CRS) secondary to septic shock and tachycardia-induced cardiomyopathy.

## CASE OUTCOME

In the ED, the patient was diagnosed with cardiorenal syndrome based on a combination of other diagnoses including severe sepsis secondary to urinary tract infection, congestive heart failure, non-ST segment elevation myocardial infarction (NSTEMI), hypertensive emergency, hypoxic respiratory failure, acute kidney injury, and polysubstance abuse including methamphetamine and marijuana. He was admitted to the ICU, and consulting specialties included neurology, cardiology, nephrology, pulmonology, and infectious disease.

The neurologist reported the patient’s acute metabolic encephalopathy was multifactorial and related to methamphetamine abuse, hyperthermia, hypoxia, and sepsis. While in the ICU, the patient self-extubated after three days and was alert and oriented to person, place, and time. Evaluation by cardiology determined that the patient suffered from hypertensive emergency with non-STEMI and was started on aspirin and a heparin drip. Transesophageal echocardiogram was negative for vegetations or valvular lesions. Left ventricular ejection fraction was 15–20% with signs of combined systolic and diastolic heart failure and was attributed to methamphetamine use. The patient required vasopressor therapy with norepinephrine and vasopressin as well as dobutamine for cardiogenic shock. The cardiologist discussed potential cardiac catheterization once stabilized; however, given global hypokinesis, it was unlikely for there to be single vessel disease.

The pulmonologist and nephrologist agreed the acute respiratory failure with hypoxia was due to pulmonary edema. After chart review, the patient was found to have acute on chronic kidney disease. The cause of this injury was likely multifactorial including CRS given the clinical picture of volume overload, tachycardia-induced cardiomyopathy from methamphetamine abuse with decreased left ventricular ejection fraction, and potential acute tubular necrosis secondary to septic shock. A furosemide trial did not produce adequate urine output, so the patient was started on sustained, low-efficiency dialysis and his respiratory status improved. Nephrology supported the diagnosis of CRS and mentioned that the patient might have a mixed picture of cardiogenic and septic shock, which would be consistent with CRS, specifically type five.

The infectious disease specialist diagnosed the patient with septic shock due to bacteremia from Gram-positive cocci in chains likely secondary to IV drug use, and initiated treatment with ceftriaxone and clindamycin. Based on leukocytosis and increasing procalcitonin, IV immunoglobulin was added for possible toxic shock syndrome secondary to group A streptococcal bacteremia. The patient responded positively to the above treatments. On repeat blood cultures, he was positive for *Enterococcus faecalis*. In addition, he had an elevated rapid plasma reagin titer consistent with late, latent syphilis. The patient ultimately left the hospital against medical advice despite extensive discussion regarding his multiple diagnoses requiring further management and care.

## RESIDENT DISCUSSION

Cardiorenal syndrome encompasses a spectrum of disorders involving both the heart and kidneys in which acute or chronic dysfunction in one organ may induce acute or chronic dysfunction in the other organ.^1^ Cardiorenal syndrome is divided into two major groups, cardiorenal and renocardiac syndromes, and five subgroups based on which organ is the cause of damage to the other organ.^2^ These hemodynamic interactions of the heart and kidney are affected by heart failure and atherosclerotic disease in both organ systems. They are also affected by chronic kidney disease (CKD) and the alterations it causes in the neurohormonal activation, cytokines, and the biochemical perturbations across the anemia–inflammation–bone mineral axis.^1^ Structural changes in the heart unique to kidney disease progression also play a role in the development of CRS.^1^

As summarized in Table 2, there are five types of CRS:^2^ CRS type 1 is characterized by acute worsening of cardiac function leading to an acute kidney injury (AKI);^3^ CRS type 2 is characterized by chronic cardiac dysfunction leading to renal dysfunction and can be used to describe chronic heart failure leading to renal failure;^3^ CRS type 3 is characterized by acute cardiac dysfunction as a result of acute renal impairment;^3^ CRS type 4 describes CKD leading to cardiac dysfunction (left ventricular failure or diastolic heart failure);^3^ and CRS type 5 is characterized by simultaneous cardiac and renal dysfunction as a part of a systemic condition whether it be acute or chronic. This most commonly includes systemic conditions such as sepsis and less so other conditions such as amyloid or vasculitis.^3^

In the acute setting, severe sepsis represents the most common and serious condition, which can affect both organs.^4^ It can induce AKI while leading to profound myocardial depression.^4^ The onset of myocardial functional depression and a state of inadequate cardiac output can further decrease renal function as discussed in type 1 CRS, and the development of AKI can affect cardiac function as described in type 3 CRS.^1^ Renal ischemia may then induce further myocardial injury in a vicious cycle injurious to both organs.^1^

To diagnose CRS, tools include biomarkers such as brain natriuretic peptide, echocardiography, and renal ultrasound. In addition, measuring central venous pressure, systolic pulmonary artery pressure, pulmonary capillary wedge pressure and left atrial pressure, and cardiac output will help in determining degree of congestion.^1^ Renal ultrasonography helps identify renal venous congestion and its clinical significance in CRS.^1^

Diuretics are key in treating CRS.^1^ However, the kidneys can stop responding due to the “braking phenomenon.”^1^ For cardiogenic shock and severe hypotensive episodes in patients with CRS, which can cause oliguria or anuria, inotropes are frequently used to improve cardiac output and renal blood flow.^5^ Historically, low-dose dopamine has been used to increase renal blood flow although there is conflicting evidence regarding its effect upon glomerular filtration rate.^5^ No clinical trial to date has demonstrated a benefit with regard to lower mortality rates.^5^ Trials of dobutamine and milrinone have shown improvement of cardiac index and, in proportion, renal blood flow; however, this has not translated into survival benefit.^5^ Treatment is directed at the prompt identification, eradication, and treatment of the source of infection while supporting organ function with invasively guided fluid resuscitation in addition to inotropic and vasopressor drug support.^6^

## FINAL DIAGNOSIS

Cardiorenal syndrome type 5.

## KEY TEACHING POINTS

- Cardiorenal syndrome is considered dysfunction in the heart or kidneys that worsens the function of the other organ. It has multiple contributing factors such as neurohormonal system activation leading to reduced forward flow and renal perfusion and increased venous pressure.^3^
- CRS is classified into two major groups, cardiorenal and renocardiac, and five subgroups based on which organ is the cause of damage to the other organ.
- To diagnose CRS, tools include biomarkers such as brain natriuretic peptide, echocardiography, and renal ultrasound as well as measuring central venous pressure, systolic pulmonary artery pressure, pulmonary capillary wedge pressure and left atrial pressure, and cardiac output.
- Early diagnosis is important, and medical treatment options are focused on improving cardiac function, reducing volume overload, and managing heart failure and chronic kidney disease.^3^

## Figures and Tables

**Table 1:** Laboratory values of a 48-year-old male with respiratory distress and altered mental status.

Laboratory name	Laboratory value	Reference ranges
Complete Blood Count		
White blood cell	16.35	4.50–11 mL
Hemoglobin	13.7	13.5–18 g/dL
Hematocrit	43.9	40.0–54.0%
Platelet count	194	130–500 K/mL
Neutrophil percentage	88.1	40.0–74.0%
Lymphocyte percentage	4.8	19.0–48.0%
Comprehensive Metabolic Panel		
Sodium	131	135–147 3.4-
Potassium	4.0	5.3 mEq/L
Chloride	96	96–108 mEq/L
Carbon dioxide	21	22–32 mEq/L
Anion gap	14	0–16
Glucose	186	< 133
Blood urea nitrogen	43	7–23 mg/dL
Creatinine	2.2	0.4–1.3 mg/dL
Estimated glomerular filtration rate (Black/African American)	38.9	≥ 60
Blood urea nitrogen/Creatinine ratio	19	7–28 Ratio
Calcium	9.1	8.6–10.5 mg/dL
Total Bilirubin	8.7	0.2–1.4 mg/dL
Aspartate aminotransferase	151	5–34 U/L
Alanine aminotransferase	47	5–50 U/L
Alkaline phosphatase	95	46–116 U/L
Total protein	7.2	6.0–8.0 g/dL
Albumin	3.8	3.5–5.0 g/dL
Miscellaneous Labs		
Lactic acid	2.6	0.5–2.2 mmol/L
Procalcitonin	125.37	<0.050 ng/ml
High sensitivity troponin #1	492.4	0–53.5 ng/ml
High sensitivity troponin #2	585.9	0–53.5 ng/ml
Myoglobin	>900	<10–110 ng/ml
Total creatine kinase	5913	46–171 U/L
Lactate dehydrogenase	457	120–246 U/L
Brain natriuretic peptide	1317.6	0–100.0 pg/ml
C-reactive protein	185.9	0–10 mg/L
International normalized ratio	1.4	0.8–1.2
Partial thromboplastin time	34.3	25.0–37.0 sec
D-dimer	1.810	<0.500 mg/ml
Fibrinogen	604	175–400 mg/dL
Salicylates	<3.0	2.0–29.0 mg/dL
Acetaminophen	4	0–199 mg/ml
Ethyl alcohol	<10	0–10 mg/dL
COVID-19 PCR	Presumptive negative	Not Detected
Arterial Blood Gas		
pH	7.49	7.35–7.45 pH
pCO_2_	27.5	35.0–45.0 mm Hg
pO_2_	75.5	80.0–100 mm Hg
HCO_3_	20.3	22.0–26.0 mEq/L
Total CO_2_	21.1	22–32 mEq/L
Oxygen Saturation	93%	94–97%
Urinalysis		
Urine protein	4+	Negative
Urine ketones	Negative	Negative
Urine blood	3+	Negative
Urine nitrite	Positive	Negative
Urine leukocyte esterase	1+	Negative
Urine red blood cells	0–4	0–4/high power field
Urine white blood cells	75–100	0–2/high power field
Urine epithelial cells	Moderate	No Units. No Range.
Urine bacteria	Marked	No Units. No Range.
Urine glucose	Negative	Negative
Urine Drug Screen		
Amphetamines	Positive	Negative
Methamphetamines	Positive	Negative
Cannabinoids	Positive	Negative

*mL,* microliters; *g,* grams; *dL,* deciliter; *K,* thousand; *mEq,* milliequivalents; *L,* liter; *mg,* milligrams; *U,* units; *mmol,* millimoles; *ng,* nanogram; *ml,* milliliters; *sec,* seconds; *COVID-19 PCR,* coronavirus disease 2019 polymerase chain reaction test; *pCO_2_,* partial pressure of carbon dioxide.

*mm Hg,* millimeters of mercury; *pO_2_,* partial pressure of oxygen; *mm Hg,* millimeters of mercury; *HCO_3_,* bicarbonate; *mEq,* milliequivalents; *L,* liter.

**Table 2:** Classification of cardiorenal syndrome based on a system proposed by Ronco and McCullough.^2^

Cardiorenal Syndrome Type	Characteristics
Type 1 (acute cardiorenal)	Acute cardiac impairment leading to acute kidney injury
Type 2 (chronic cardiorenal)	Chronic cardiac impairment leading to renal impairment
Type 3 (acute renocardiac)	Acute kidney injury leading to cardiac impairment
Type 4 (chronic renocardiac)	Chronic kidney disease leading to cardiac impairment
Type 5 (secondary cardiorenal)	Systemic condition leading to both cardiac and renal impairment

## References

[1] Rangaswami J, Bhalla J, Chang T (2019). Cardiorenal Syndrome: Classification, Pathophysiology, Diagnosis, and Treatment Strategies: a scientific statement from the American Heart Association. Circulation.

[2] Ronco C, McCullough P, Anker S (2010). Cardio-renal syndromes: report from the Consensus Conference of the Acute Dialysis Quality Initiative. Eur Heart J.

[3] Hadjiphilippou S, Kon S (2016). Cardiorenal syndrome: review of our current understanding. J R Soc Med.

[4] Ronco C, Di Lullo L (2014). Cardiorenal syndrome. Heart Fail Clin.

[5] Shah BN, Greaves K (2010). The cardiorenal syndrome: a review. Int J Nephrol.

[6] Ronco C, Haapio M, House A (2008). Cardiorenal syndrome. J Am Coll Cardiol.

